# Demographic Processes Underlying Subtle Patterns of Population Structure in the Scalloped Hammerhead Shark, *Sphyrna lewini*

**DOI:** 10.1371/journal.pone.0021459

**Published:** 2011-07-14

**Authors:** Holly A. Nance, Peter Klimley, Felipe Galván-Magaña, Jimmy Martínez-Ortíz, Peter B. Marko

**Affiliations:** 1 Department of Biological Sciences, Clemson University, Clemson, South Carolina, United States of America; 2 Department of Wildlife, Fish, and Conservation Biology, University of California Davis, Davis, California, United States of America; 3 Centro Interdisciplinario de Ciencias Marinas (CICIMAR-IPN), La Paz, Mexico; 4 Escuela de Pesca del Pacífico Oriental (EPESPO), Manta, Ecuador; University of Canterbury, New Zealand

## Abstract

Genetic diversity (θ), effective population size (N_e_), and contemporary levels of gene flow are important parameters to estimate for species of conservation concern, such as the globally endangered scalloped hammerhead shark, *Sphyrna lewini*. Therefore, we have reconstructed the demographic history of *S. lewini* across its Eastern Pacific (EP) range by applying classical and coalescent population genetic methods to a combination of 15 microsatellite loci and mtDNA control region sequences. In addition to significant population genetic structure and isolation-by-distance among seven coastal sites between central Mexico and Ecuador, the analyses revealed that all populations have experienced a bottleneck and that all current values of θ are at least an order of magnitude smaller than ancestral θ, indicating large decreases in N_e_ (θ = 4N_e_μ), where μ is the mutation rate. Application of the isolation-with-migration (IM) model showed modest but significant genetic connectivity between most sampled sites (point estimates of Nm = 0.1–16.7), with divergence times (t) among all populations significantly greater than zero. Using a conservative (i.e., slow) fossil-based taxon-specific phylogenetic calibration for mtDNA mutation rates, posterior probability distributions (PPDs) for the onset of the decline in N_e_ predate modern fishing in this region. The cause of decline over the last several thousand years is unknown but is highly atypical as a post-glacial demographic history. Regardless of the cause, our data and analyses suggest that *S. lewini* was far more abundant throughout the EP in the past than at present.

## Introduction

Modern fishing practices have led to declines in numerous marine species [1]–[3], with long-lived fish and mammals particularly susceptible to over-harvesting [4], [5]. Among the most affected and ecologically important species are sharks [6], [7], which sit atop marine food webs, providing significant top-down control over many other pelagic and benthic marine species [8]. Due to declines in other fin-fishes and the high demand for shark fins [9], [10], sharks are among the most sought-after harvested marine species. At the same time, however, sharks (particularly large sharks), remain highly enigmatic, with relatively little known about their population structure, life-histories, and recent demographic histories in comparison to analogous apex predators on land, but see [11].

Mark-and-recapture studies have figured prominently in estimating long-range movements, behaviors, and survival in sharks [12]. In many cases, genetic data have been collected from threatened or declining marine species [13], [14] with the idea that those data will provide information about important demographic parameters and processes relevant to conservation, like genetic diversity (θ), effective population size (N_e_), and interpopulation connectivity [15]. For the 11 largest or “great” species of sharks, population genetic data are particularly limited, with the majority having been collected over large geographic scales with an analytical focus on global phylogeography and delineation of evolutionary distinct units (ESUs) for conservation [16]–[20]. We have therefore used a combination of classical and coalescent population genetic methods to reconstruct the regional demographic history of the IUCN globally endangered scalloped hammerhead shark, *Sphyrna lewini*, across its Eastern Pacific (EP) range. *S. lewini* is a large, highly-mobile circumtropical marine predator found along continental margins and oceanic islands [21] that forms large and conspicuous aggregations, particularly in the tropical EP [22]–[24]. This shark is caught both intentionally and as by-catch throughout its range [7] and Western North Atlantic stocks alone have experienced an estimated 83% reduction between 1981 and 2005 [25]. Previous genetic work on *S. lewini* has yielded estimates of population structure, female effective population size (N_ef_) and gene flow that vary widely among different regions across the globe [17], [26], [27]. Although some of this variability could be real, contrasting patterns among recent studies could also reflect a combination of significant differences in the spatial scale of analysis, large differences in sample sizes (of individuals), and the predominant use of only a single (mtDNA) locus.

To reconstruct the demographic history of *S. lewini*, detect changes in N_e_, and estimate levels of contemporary gene flow, we have used a combination of mtDNA sequences and 15 microsatellite loci. Currently, only the isolation-with-migration (IM) class of models [28]–[31] lacks the assumption that gene flow and genetic drift are in an evolutionary equilibrium, and have therefore become valuable tools for disentangling the effects of ancestral polymorphism and contemporary gene flow in a statistically robust way. These analytical methods allowed us to consider patterns of genetic differentiation from a temporal perspective and delineate current populations for our estimates of N_e_, as well as estimate change in N_e_ over time to help interpret levels of genetic diversity. Low diversity has been found in several species of sharks [32]–[35] and therefore may be common in this group, or alternatively, could be the result of population decline.

## Methods

### Ethics Statement

No ethical or institutional approval was required for the field-based zoological and genetical research described in this paper. No live specimens were obtained or used.

### Sampling, DNA extraction, sequencing, and genotyping procedures

**Figure 1:**
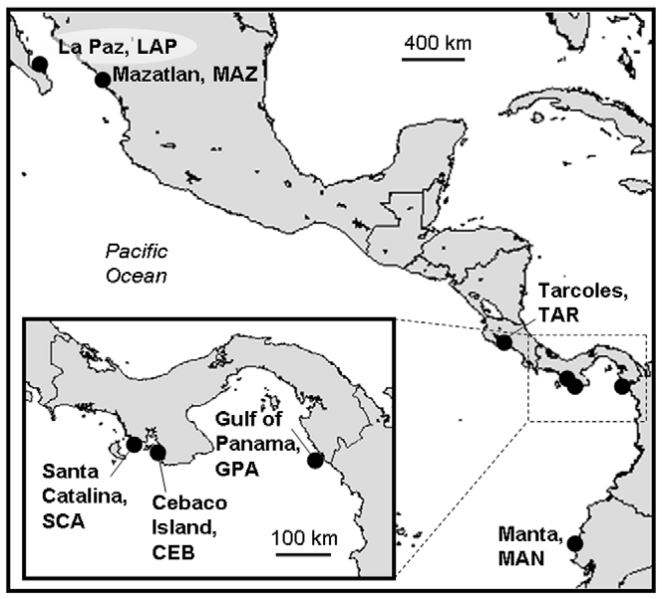
Map of Eastern Pacific range of *Sphyrna lewini* and study area. Sample localities and their associated abbreviations indicated by black dots. The three Panamanian sites are enlarged due to their close proximity to one another.

We collected 221 tissue samples from artisanal fishers at six Eastern Pacific sites between 2007 and 2008 (Fig. 1, Table S1, Table S2). With the exception of Manta, Ecuador, all samples came from sharks caught in close proximity to the fish camps where they were collected (<40 km from shore [36]); samples collected in Manta were caught farther off shore, between mainland Ecuador and the Galapagos Islands. This likely explains why Manta was the only sample with adults; all other samples were comprised of 1–3 year old juveniles. Samples were stored in 90% ethanol and genomic DNA was isolated with proteinase K tissue digestion in 2× CTAB, followed by two chloroform∶isoamyl alcohol (24∶1) extractions and precipitation with ethanol. DNA was dried, re-suspended in 50 µL water, and frozen.

We amplified and scored 15 microsatellite loci from all 221 individuals. Thirteen were developed for *S. lewini* [37] and two (Cli-12 and Cli-100) for the blacktip shark [38]. All PCR reactions [37] were conducted using a DNA Engine DYAD Peltier Thermal Cycler (MJ Research, Inc.) and visualized on an ABI 3130 (Applied Biosystems, Inc.) sequencer. We scored individual genotypes with GeneMapper v. 3.7 (Applied Biosystems, Inc.).

We also sequenced a 548 bp fragment of the mtDNA control region from 126 individuals with the Pro-L and SLcr-H primers [17] using the following cycling temperature profile: 95°C for 4 minutes, 40 cycles of 95°C for 1 minute, 57°C for 1 minute, slow ramp (1°C/s) to 72°C for 1 minute, 30 seconds, followed by an extension at 72°C for 10 minutes. Each reaction contained 1× GoTaq buffer, 0.16 µM Pro-L primer, 0.16 µM SLcr-H primer, 0.1% Triton X-100, 1.25 mM dNTPs, 0.7 U GoTaq polymerase (Promega), and 0.5 µl genomic DNA, in a total volume of 25 µl. Because all of the informative sites were at one end of the fragment [17], we only sequenced with the Pro-L primer. However, any chromatograms with ambiguous base calls were also sequenced in the opposite direction with the SLcr-H primer. Sequences were visualized on an ABI 3130 sequencer, chromatograms edited with Sequencher v.4.2.2 (Gene Codes Corp.), aligned using CLUSTAL-X v.1.81 [39], and checked by eye.

### Microsatellite and MtDNA diversity

Microsatellite loci were checked for evidence of nulls using MICRO-CHECKER v. 2.2.3 [40], tested for linkage disequilibrium and deviations from Hardy-Weinberg equilibrium (HWE) with ARLEQUIN v. 3.11 [41], and effective numbers of alleles (*A_E_*) per sample were calculated in Genalex [42]. For the mtDNA, we calculated Fu's *F_s_* [43] in ARLEQUIN, and Fu and Li's D* [44] in DNAsp v. 4.90 [45] using 10,000 simulations (conditional on θ).

### Kinship Analyses

Because of the high number of juveniles, we estimated relatedness among individuals within samples using COLONY2 [46], which searches for the maximum likelihood configuration of sibship assignments for all individuals in a sample based on microsatellite genotypes. We then calculated the percentage of half- or full-sibling pairings with ≥95% probability. Low haplotype diversity prohibited the identification of maternal siblings based on mtDNA. Because COLONY2 will overestimate the proportion of siblings when sample sizes are small with respect to the total population [47], [48], as a control comparison, we also estimated the proportion of half- and full-siblings among the combined La Paz and Tarcoles samples. These two nurseries are separated by over 3000 km of coastline, so we expected sibling pairs between these two sites to be much less frequent than within an individual site if sibship estimates within each sample were meaningful.

### Genetic structure

For the microsatellites, we estimated F_ST_ and R_ST_ among sites with a locus-by-locus Analysis of Molecular Variance (AMOVA) in ARLEQUIN. Confidence intervals for F_ST_ were generated by bootstrapping over loci (20,000 replicates). For the mtDNA, we created a haplotype network with statistical parsimony in TCS v.1.21 [49], [50]. We then used MODELTEST v. 3.8 [51] to identify the best-fitting substitution model [HKY model selected over HKY+G (LLR = 2.62, *P* = 0.05) and HKY+I (LLR = 5.08, *P* = 0.01)]. We then estimated F_ST_ and Φ_ST_, the latter with an AMOVA using the best-fitting model available in ARLEQUIN [52].

Although population differentiation was low, we also used STRUCTURE v. 2.3.3 [53] to infer the number of discrete populations. We set K = 1–20 for each run, assuming prior population information and an admixture model allowing for mixed ancestry of individuals [54]. Each run was repeated three times with independent allele frequencies, 100,000 steps, and a burn-in of 10,000 steps.

### Demographic analyses

We used several different methods to investigate past changes in N_e_, utilizing approaches that employ different assumptions and different combinations of the nuclear and cytoplasmic markers. First, we conducted mtDNA mismatch analyses in ARLEQUIN under a model of sudden demographic expansion. To determine how well the sudden expansion model fit our data, we calculated Harpending's raggedness index, *r* [55] and assessed the significance of *r* with 1000 parametric bootstrap replicates. For samples not deviating significantly from the expansion model, we then estimated the time since the start of expansion using the formula τ = 2 µt, where t is the number of years since expansion and μ is the per locus, per year mutation rate. Confidence intervals for τ were estimated with 1000 parametric bootstrap replicates.

We then calculated the M-ratio statistic with the software *M_P_val* [56] to test for evidence of a recent population bottleneck in each sample of microsatellites. The empirical value of M was compared to a simulated equilibrium distribution based on the two-phase model of microsatellite mutation. This simulated critical value (M*_C_*) was calculated with 10,000 replicates in *critical_M* [56]. We analyzed our data using two different values for p_s_, the percentage of mutations that follow the single-step mutation model, and Δ_g_, the mean size of larger mutations; p_s_ = 0.88 and Δ_g_ = 2.8, and then more conservative values p_s_ = 0.90 and Δ_g_ = 3.5 [56]. Because empirical values of M were equal for both, we only showed M-ratios calculated for the latter p_s_ and Δ_g_ values.

To test for significance of the M-ratios, we used a range of values for pre-bottleneck θ = 4N_e_μ (0.01, 0.1, 1.0, 10.0), yielding pre-bottleneck N_e_ of 250, 2500, 25,000, and 250,000, respectively. To calculate θ for these tests, we chose a microsatellite mutation rate (μ) of 1×10^−5^, which is the slower end of the range estimated in mammals [57]–[60], since mitochondrial and nuclear markers mutate roughly an order of magnitude slower in sharks than in mammals [61], [62]. We used values of p_s_ = 0.90 and Δ_g_ = 3.5 to calculate M*_C_* [56]. Because outlier alleles and violations to the single-step mutation (SSM) model can bias values of M [56], we ran all tests with the full data set, and then re-ran tests after removing outlier alleles (those at the ends of the size range that were separated by more than 10 bp from the next allele) and loci that had at least one allele not conforming to a di-nucleotide repeat pattern.

We also tested for evidence of a recent reduction in N_e_ in the microsatellite dataset with BOTTLENECK [63] using the Wilcoxon sign-rank test under the infinite alleles model (IAM), the two-phase model (TPM), and the single-step model (SSM), given that all of the loci we developed had point mutations and therefore did not conform to the strict SSM. Changes in N_e_ were also estimated with MSVAR v. 1.3 [64], which applies MCMC simulations of the mutation-coalescent history to present day genotypes in a sample by characterizing the posterior distribution of the parameters N_0_ (current population size), N_1_ (ancestral population size), μ (mean mutation rate of all loci), and t (time since population size change) for each population (GPA was too small for this analysis). We varied priors for each locus for N_0_, N_1_, μ, and t, [64]. We chose a range of microsatellite mutation rates (μ), as recommended by the authors [64], between 1.0×10^−5^ and 2.0×10^−4^, given that, 1) the estimated microsatellite μ is 1.5×10^−4^ in zebra fish [65] and 5.56×10^−4^ in the common carp [66], and 2) the range of μ in mammals is 10^−5^ to 10^−2^ [67] and both mtDNA and nuclear markers mutate roughly an order of magnitude slower in sharks than in mammals [61], [62]. Prior values were updated throughout the analysis, and modeled with an exponential change in population size. Each run was 200 million steps, with a burn-in of 10,000 steps and output every 10,000 steps. We used TRACER v. 1.4.1 [68] to graph posterior distributions of N_0_, N_1_, μ, and t, and to calculate the 95% mean probability densities of each parameter.

Although age at first reproduction (roughly 15 years in *S. lewini* [53], [54]) is typically used as a proxy for generation time [17], coalescent estimates of N_e_ require an estimate of G, the average age of breeding adults [69]. To estimate G, we used a method [69] requiring life history data, and since none exists for EP populations, we used survival rates for *S. lewini* in the East Atlantic [70]. For age specific reproductive rates, we used 15 years as the age of first reproduction [71], [72], and a mean litter size of 23 pups, which remains relatively constant throughout adulthood (Nguyen and Piercy unpub. data). From these data, we calculated l*_i_* (age-specific survival rates), b*_i_* (birth rates), and p*_i_* (probability of a gene being inherited from a parent of age *i*), for all age classes, *I* [69], and then used these data to calculate G (Table S3). We used MATLAB v. 2007a on a Windows XP operating system to calculate mean generation time (G) based on the equation of Felsenstein [73].

### Population divergence times and migration rates

We estimated genetic diversities (θ_1_, θ_2_, and ancestral θ_A_), migration rates (*m_1_* and *m_2_*), and time since population divergence (*t*) for all pairs of samples using the program IMa [29] on the CBSU computing clusters at Cornell University. The “isolation with migration” model in IMa does not assume gene flow and genetic drift are in equilibrium, making it most appropriate for recently diverged populations that share haplotypes and alleles due to both gene flow and ancestral polymorphism. Although IMa2 [74] can handle multiple populations at once, we analyzed all possible pairs of populations separately in IMa because IMa2 requires a well-supported phylogeny of the groups of individuals being analyzed [74].

We started with analyses in “MCMC Mode” (M-Mode) using the full complement of model parameters (i.e., θ_1_≠θ_2_≠θ_A_, and *m_1_*≠*m_2_*), with broad priors for all, reducing them in repeated runs to more densely sample the posterior distribution. Once several replicates converged on the same answer, we used the saved genealogies from three separate M-Mode runs in a new analysis using the nested models option in “Load Trees Mode” (L-Mode) to determine if the fully parameterized IMa model was a significantly better fit to the data than a series of simpler models with fewer parameters, based on log-likelihood ratio tests [29]. We converted migration parameters *m_1_* and *m_2_* into the number of migrants per generation (Nm) using the equation Nm = (θ *m*)/4.

To convert divergence times from IMa scaled by mutation (*t*/μ) into units of years, μ of at least one locus must be known. Given that microsatellite mutation rates are unknown for sharks and can vary by an order of magnitude within individual species [75], we used only mtDNA substitution rates calculated specifically for hammerhead sharks (below) and allowed IMa to infer separate rate scalars for the microsatellite loci [76]. To estimate the substitution rate of mtDNA control region, we built a phylogeny for all eight sphyrnid species using BEAST v. 1.5.4 [77] based on previously published [78] nuclear (ITS2, Dlx1, and Dlx2) and mitochondrial (NADHd2, D-loop, Cyt-b, and CO1) genes. Using *Carcharhinus acronotus* as the outgroup [78], we chose lognormal priors for a 40.4 (+/−1.05) million years (my) divergence time between *Carcharhinus* and Sphyrnidae [79], and a 21.5 (+/−1.05) my divergence time for species within Sphyrnidae (based on the first sphyrnid in the fossil record [79]) to calibrate the substitution rate for each gene. Five runs, totaling 225 million MCMC steps resulted in a divergence rate of 1.21% per million years (my) for Sphyrnidae D-loop, corresponding to a mutation rate of 6.03×10^−9^ substitutions per site per year. This is only slightly faster than a divergence rate of 0.8% per my, which was based on the assumption that Western Atlantic and Eastern Pacific populations of *S. lewini* separated three million years ago by the Isthmus of Panama [17].

## Results

### Microsatellite and mtDNA diversity

Average observed and expected heterozygosity across all loci and populations were 0.770 and 0.792, respectively (Table S1), and across all loci in all populations, the number of effective alleles (*A_E_*) was distinctly less than the total number of alleles (Table S1). After Bonferroni correction of alpha [80], four loci deviated significantly from HWE in one or two populations (Table S1). Two loci (Cli-12 and Cli-100) were in linkage disequilibrium in two of seven samples. Micro-Checker showed five loci had no nulls in any of the samples, and ten had potential nulls in one or two samples.

**Figure 2:**
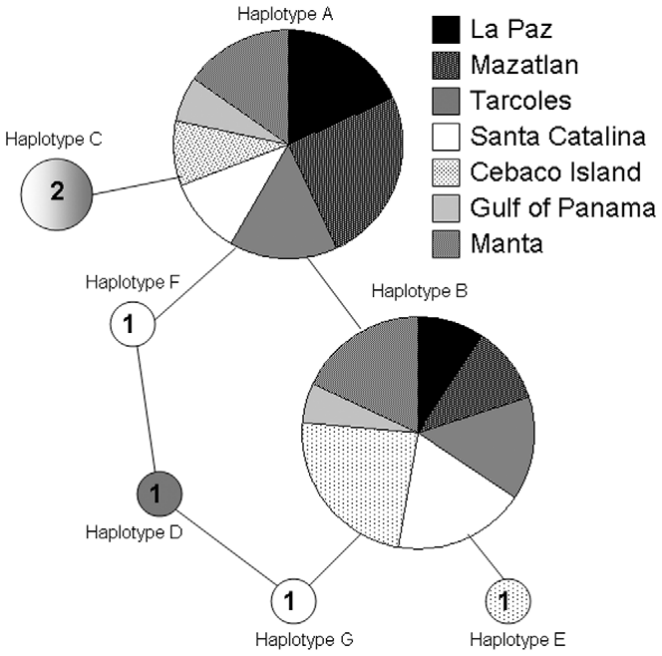
Haplotype network showing proportion of haplotypes per population. Haplotypes A and B are common to all populations. Haplotype C is shared by TAR and SCA (hence, the two shades), haplotypes D and E are unique to TAR and CEB, respectively, and haplotypes F and G are unique to SCA. Numbers inside haplotypes C through G indicate the number of haplotypes present in our sampled individuals.

As with previous analyses of mtDNA in the Pacific, we found low levels of diversity in the EP: seven mtDNA control region haplotypes that differed by a maximum of two base pair changes. Haplotypes A and B were common to all locations, C and D were found in one to two locations, respectively (Fig. 2). D and E were novel to this study (GenBank accession numbers HQ916311 and HQ916312, respectively). Fu's *F_s_* was positive for each sample, though none were significant (Table S2). Fu and Li's D* was negative for three samples: TAR, SCA and CEB, though none were significant (Table S2).

### Relatedness

We estimated that only 3.7% of individuals per sample were members of a full-sibling pair. Half-sibs were more frequent, with a mean of 59.2% of individuals being half-sibs. However, when we estimated sibship in the La Paz and Tarcoles samples combined (two sites separated by more than 3000 km), we found similar proportions of siblings: 7.8% and 59.4% for full- and half-sib pairs, respectively, suggesting our sample sizes are too small to recover meaningful estimates of kin. All proportions of siblings reported were recovered with a likelihood >95%.

### Genetic structure

For the microsatellites, all R_ST_ estimates were insignificant (not shown), but the global estimate of F_ST_ was highly statistically significant (F_ST_ = 0.005, *P*<0.001; see Table 1). Pairwise estimates of F_ST_ (ranging from 0.015 – 0.002; Table 2) were also significant for most comparisons. Correlation between geographic distance and genetic differentiation was marginally insignificant for F_ST_ (r = 0.302, *P* = 0.063) and marginally significant for R_ST_ (r = 0.422, *P* = 0.032).

**Table 1:** AMOVA results for all sites, characterizing spatial structure with both mtDNA (Φ_ST_) and microsatellites (F_ST_).

Marker	Source of variation	d.f.	SS	Variance components	Φ_ST_/F_ST_
mtDNA	among pops	6	2.746	Va 0.009	0.031
	within	119	34.57	Vb 0.291	
msats	among pops	6	43.293	Va 0.024	0.005*
	within	599	3147.3	Vb 5.254	

*indicates significant at α = 0.05.

**Table 2:** Pairwise locus-by-locus AMOVA results characterizing structure based on microsatellites (F_ST_) between all Eastern Pacific sites.

	LAP	MAZ	TAR	SCA	CEB	GPA	MAN
**LAP**	*						
**MAZ**	0.000	*					
**TAR**	**0.010**	**0.007**	*				
**SCA**	0.005	**0.005**	**0.007**	*			
**CEB**	**0.015**	**0.012**	**0.013**	**0.011**	*		
**GPA**	0.012	**0.014**	0.009	0.005	0.000	*	
**MAN**	**0.006**	**0.004**	**0.009**	**0.002**	**0.007**	0.009	*

Values significant at α = 0.05 are indicated in bold.

For the mtDNA sequences, neither F_ST_ nor Φ_ST_ across all sites were statistically significant (only Φ_ST_ values shown in Table 1) and no pairwise F_ST_ estimates were significant. However, pairwise estimates of Φ_ST_ were significant between one central Panama sample (SCA) and both Mexico samples (SCA-LAP Φ_ST_ = 0.17, *P* = 0.03 and SCA-MAZ Φ_ST_ = 0.21, *P* = 0.01). The Mantel test showed a marginally significant correlation between Φ_ST_ and geographic distance (r = 0.523, *P* = 0.039), though no correlation was detected with the frequencies of haplotypes (F_ST_).

STRUCTURE showed that K = 1 had the highest probability, indicating no differentiation among samples (plots of assignment for K = 1 to 20 showed no evidence of subdivision, not shown). With such low F_ST_ estimates, however, STRUCTURE v. 2.3.3 is not expected to be informative [53].

### Demographic analyses

MtDNA mismatch distributions showed evidence of relatively ancient demographic expansions across all populations (Table 3): the model of sudden demographic expansion was only rejected for MAN. For all populations, the modal number of nucleotide differences between haplotypes peaked between zero and one (graphs not shown), indicating relatively recent expansions. After conversion with the mtDNA substitution rate from BEAST, point estimates of time since expansion among all populations (excluding MAN) were between 90,606 and 136,061 years ago (Table 3).

**Table 3:** Mismatch distribution results.

Sample	Coordinates	*τ*	90% CI	*r*	*t* (years)	90% CI *t* (years)
**LAP**	N 24.20, W 110.40	0.641	0.042–1.277	0.208	97,121	6364–193,485
**MAZ**	N 23.20, W 106.40	0.598	0.105–1.191	0.201	90,606	15,909–180,455
**TAR**	N 9.80, W 84.80	0.898	0.336–1.617	0.140	136,061	50,909–245,000
**SCA**	N 7.56, W 81.30	0.867	0.375–1.578	0.149	131,364	56,818–239,091
**CEB**	N 7.55, W 81.00	0.812	0.281–1.559	0.201	123,030	42,576–236,212
**GPA**	N 7.01, W 78.19	0.898	0–22.75	0.347	136,061	0–3,446,970
**MAN**	S 1.10, 84.95	0.814†	0.313–1.414	0.280	NA	NA

†indicates significance at α = 0.05.

Tau (τ) and 90% confidence intervals of simulations under the model of sudden expansion for each population are shown. Harpending's raggedness index (*r*), time since population expansion in years (*t*), and associated 90% confidence intervals are shown for all populations where the sudden expansion hypothesis could not be rejected. Time since expansion (*t*) was estimated using the fossil-calibrated substitution rate (6.03×10^−9^ subs/yr). Site abbreviations correspond to locations in Figure 1.

**Figure 3:**
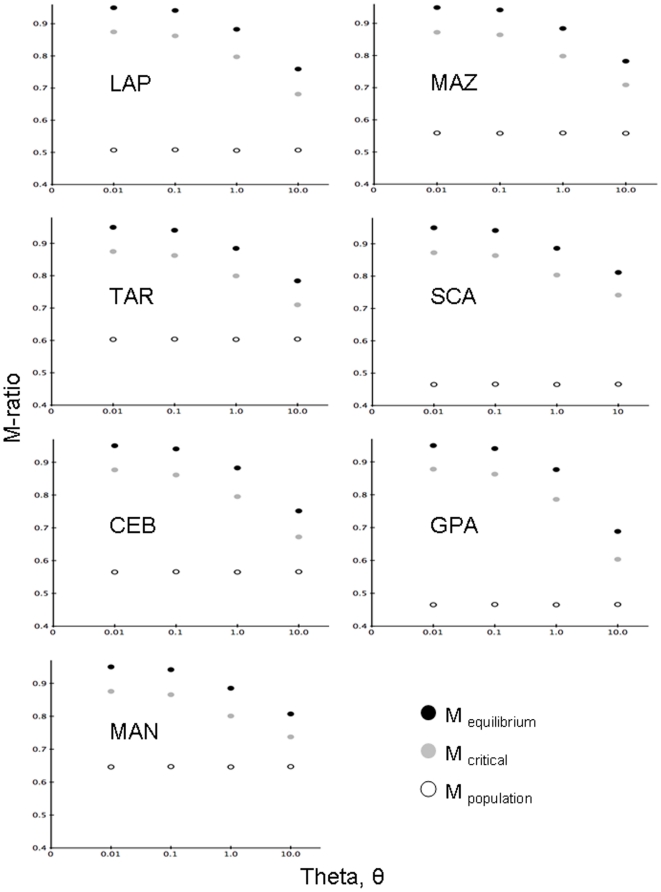
M ratio test results based on microsatellite data for each population. The population-specific M ratio (open circles), average M from simulations assuming each population is in drift-mutation equilibrium (black circles), and critical M*_c_* based on these simulations (gray circles) are shown. M values below M*_c_* indicate a population has undergone a recent bottleneck. All data shown here were calculated with a proportion of single step mutations (p_s_) of 0.90 and an average size of mutations evolving more than one repeat unit (Δ_g_) of 3.5. All M values were calculated with θ = 0.01, 0.1, 1.0, and 10.0, corresponding to N_e_ = 1445, 14,451, 144,509, and 1,445,087, respectively.

Using the full microsatellite data set, the M-ratio for each population was lower than the simulated critical M*_C_* value for each value of θ (Fig. 3) providing evidence of recent population declines. Removal of outlier alleles and loci not conforming to the di-nucleotide repeat pattern (see Methods), resulted in only the Manta population lacking evidence of decline, and only when tested with a pre-bottleneck value of θ = 10 (M_MAN_ = 0.845). Results from BOTTLENECK depended on the model of microsatellite mutation: under the IAM model alone, the results showed a significant excess in gene diversity, and therefore a recent bottleneck, for all populations (p-value for one-tail test<0.05), except Cebaco Island (CEB) and Manta (MAN).

MSVAR also showed population declines, showing that current N_e_ at each sample site was at least two orders of magnitude smaller than historic N_e_, with point estimates of the onset of decline ranging between 3600 and 12,000 years ago (Table 4). Results from IMa (below) also showed that current θ is smaller than ancestral θ_A_ by 1–3 orders of magnitude (Table 5, 6). Although 95% posterior probability densities (PPDs) of these estimates were wide, both coalescent methods show significant decline, with no overlap between most (23 of 30 from IMa and 3 of 6 from MSVAR) 95% PPDs of current and ancestral N_e_.

**Table 4:** Results from MSVAR (Beaumont 1999) analyses using only microsatellite data.

Population	N_e0_	N_e1_	t (in years)
**LAP**	435.51	39,627.80	8452.79
**95% HPD**	(36.16–4717.37)	(4718.46–324,041.03)	(493.06–117,733.49)
**MAZ**	384.68	43,551.19	6181.59
**95% HPD**	(28.89–4627.01)	(4927.20–365,426.47)	(386.99–81,320.49)
**TAR**	481.95	34,994.52	5766.34
**95% HPD**	(49.57–4607.87)	(4102.99–289,867.82)	(347.46–86,616.37)
**SCA**	284.32	39,728.30	5870.84
**95% HPD**	(28.66–2777.15)	(4822.80–326,061.90)	(562.99–59,278.88)
**CEB**	226.67	38,256.04	3639.15
**95% HPD**	(8.00–4952.22)	(4463.75–333,042.76)	(116.33–79,031.46)
**MAN**	604.09	35,958.37	11,917.91
**95% HPD**	(50.14–6428.36)	(4303.28–296,619.70)	(830.42–145,378.40)

Current (N_e0_) and historic (N_e1_) estimates of effective population size, and time in years (t) since the onset of population decline are shown. All point estimates are followed by 95% highest posterior density intervals, as calculated in Tracer v. 1.4.1 [51].

**Table 5:** IMa results.

Samples	θ_1_	θ_2_	θ_A_	*m_1_*	*m_2_*	t/μ	t, years
**LAP-MAZ**	0.04	0.40	87.50	184.40	35.55	0.006	51.16
**95% HPD**	(0.01–4.00)	(0.08 - ∞)	(51.70–129.70)	(96.4 - ∞)	(13.05 - ∞)	(0.005–0.110)	(38.17–895.77)
**LAP-TAR**	0.11	0.37	74.69	51.03	9.00	0.010	243.45
**95% HPD**	(0.06–3.84)	(0.17–5.00)	(48.07–138.05)	(25.68–484.58)	(1.56–202.20)	(0.008–0.217)	(186.73–5117.27)
**LAP-SCA**	0.04	0.17	31.73	105.00	15.95	0.003	159.50
**95% HPD**	(0.03–0.87)	(0.07–8.39)	(23.54–58.28)	(33.0–1079.40)	(7.15–894.85)	(0.001–0.033)	(66.0–1831.50)
**LAP-CEB**	0.02	0.79	49.21	23.40	85.25	0.004	86.53
**95% HPD**	(0.02–0.99)	(0.29 - ∞)	(33.61–83.14)	(21.0–1107.0)	(23.65–987.25)	(0.003–0.099)	(55.07–1945.03)
**LAP-MAN**	0.02	0.18	36.55	170.1	32.80	0.002	36.55
**95% HPD**	(0.01–0.59)	(0.08 - ∞)	(24.95–69.45)	(90.90 - ∞)	(26.40–1469.6)	(0.001–0.022)	(24.95–69.45)
**MAZ-TAR**	0.05	1.15	89.55	57.05	14.63	0.012	287.15
**95% HPD**	(0.02–1.76)	(0.09 - ∞)	(56.85–150.45)	(16.45–353.85)	(0.23–449.78)	(0.008–0.314)	(187.27–7828.00)
**MAZ-SCA**	0.04	0.16	35.6	95.4	67.65	0.002	124.36
**95% HPD**	(0.02–0.58)	(0.05–1.85)	(26.51–71.87)	(40.20 - ∞)	(12.65–805.75)	(0.001–0.027)	(46.64–1399.09)

**Table 6:** IMa results (continued).

Samples	θ_1_	θ_2_	θ_A_	*m_1_*	*m_2_*	t/μ	t, years
**MAZ-CEB**	0.02	0.14	43.19	127.00	82.45	0.002	35.64
**95% HPD**	(0.01–0.40)	(0.07 - ∞)	(29.47–74.55)	(63.0 - ∞)	(31.45–1481.55)	(0.001–0.025)	(17.82–447.24)
**MAZ-MAN**	0.01	0.13	32.83	241.5	145.21	0.001	13.83
**95% HPD**	(0.01–0.23)	(0.05–13.70)	(21.0–57.40)	(151.5 - ∞)	(55.26–2198.64)	(0.001–0.009)	(6.38–98.92)
**TAR-SCA**	0.61	1.26	85.41	30.38	4.42	0.029	19,968.14
**95% HPD**	(0.13–2.13)	(0.33–3.01)	(47.90–132.84)	(6.83 - ∞)	(0.05–41.09)	(0.021–0.377)	(14,363.05–263,789.59)
**TAR-CEB**	0.05	0.55	64.4	29.75	52.88	0.010	375.7
**95% HPD**	(0.05–1.88)	(0.47–19.45)	(35.76–127.12)	(14.75–450.75)	(10.58–420.98)	(0.007–0.276)	(261.2–109,26.87)
**TAR-MAN**	0.01	0.09	44.14	162.35	41.3	0.003	88.32
**95% HPD**	(0.01–0.30)	(0.06–6.39)	(31.53–86.13)	(75.65 - ∞)	(21.70–1305.50)	(0.001–0.023)	(37.37–777.91)
**SCA-CEB**	0.02	0.09	41.52	74.75	78.75	0.002	110.02
**95% HPD**	(0.01–0.25)	(0.02 - ∞)	(29.36–62.16)	(12.65 - ∞)	(1.05–1288.35)	(0.000–0.017)	(18.34–1026.84)
**SCA-MAN**	0.07	0.35	88.27	81.25	15.23	0.017	513.52
**95% HPD**	(0.06–2.73)	(0.29–11.42)	(50.83–173.03)	(37.25–455.75)	(6.13–331.63)	(0.012–0.212)	(348.35–6357.42)
**CEB-MAN**	0.20	0.53	83.07	66.75	19.21	0.057	857.77
**95% HPD**	(0.05 - ∞)	(0.11–8.30)	(58.11–122.07)	(14.75–241.75)	(0.115–166.64)	(0.019–0.226)	(280.86–3423.5)

θ = 4N_e_μ for populations 1, 2, and the ancestral population from which they arose, migration parameters *m_1_* and *m_2_*, and time in years (t) since populations diverged using the fossil-calibrated substitution rate (6.03×10^−9^ subs/year) are shown. 95% HPDs (highest probability densities) represents the interval on the x-axis where 95% of the area under the posterior probability density curve lies. Upper boundaries of ∞ indicate the HPD had not yet reached zero, though was approaching it. In each pair of populations, population 1 is listed first.

### Population divergence times and migration

**Figure 4:**
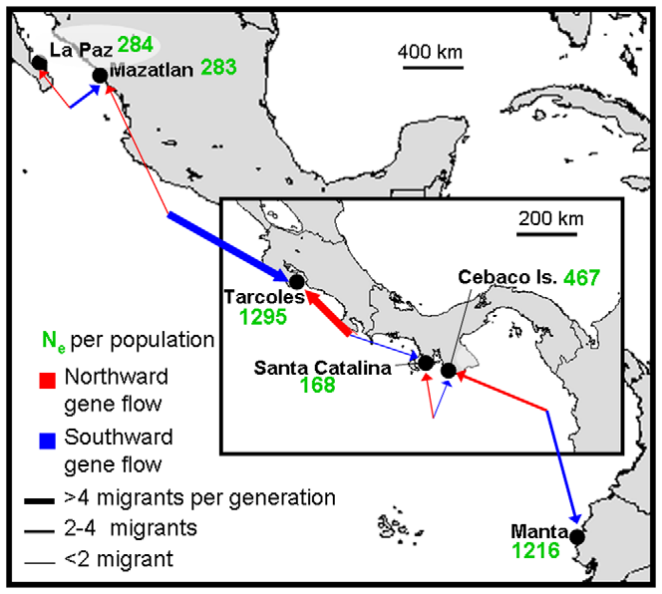
Map showing relative migration rates (Nm) between only adjacent pairs of EP populations. Nm refers to the number of migrants per generation. Red arrows indicate northward gene flow; blue indicate southward flow. Thickness of arrows corresponds to magnitude of flow, or number of migrants per generation. Values in green indicate current N_e_, as averaged from estimates of MSVAR and IMa. N_e_ from IMa was calculated with the equation θ = 4N_e_μ.

For each comparison of adjacent samples, simpler demographic models in IMa were rejected in favor of the fully parameterized model (Table S4). Estimates of *t* for all population pairs were significantly greater than zero, given that the posterior probability distributions (PPDs) drop to zero as *t* approaches zero. Using our conservative fossil-based estimate of the mtDNA control region mutation rate from BEAST, 95% PPDs were large for all estimates of t, although the majority (13 of 15) were completely contained within the Holocene (Table 5, 6). The posteriors for migration showed that gene flow was also greater than zero among all comparisons. Maximum likelihood estimates (MLEs) of Nm = θ*m*/4 (the number of migrants per generation) between all possible population pairs ranged between 0.1 and 16.7 (Fig. 4).

## Discussion

### Current and ancestral population sizes

The most consistent and statistically significant result from our analyses of 15 microsatellite loci and mtDNA control region sequences from *S. lewini* was that current population size (N_e_) is substantially (1–3 orders of magnitude) smaller than ancestral N_e_ among all Eastern Pacific (EP) sites studied, indicating that the demographic history of *S. lewini* in the EP is marked by statistically significant declines in N_e_. Although 95% posterior probability distributions (PPDs) from both MSVAR (microsatellites) and IMa (microsatellites and mtDNA) were wide, the majority of the credibility intervals for current and ancestral estimates of N_e_ from IMa (23 of 30 comparisons) and MSVAR (3 of 6 comparisons) did not overlap, and where there was overlap among the three MSVAR comparisons, it was less than 5% of the area under the curves. Though we varied the priors for the microsatellite mutation rate (μ) of each locus (as recommended for MSVAR [64]), the range (μ between 1.0×10^−5^ and 2.0×10^−4^) included rates for bony fish [65], [66] and the slower end of known rates for mammals [67]. Whether our range of prior μ is too fast or too slow, error in the estimation of μ by the MSVAR method will not change the fact that current and ancestral estimates of N_e_ in general do not overlap – a faster or slower μ would bias the two parameters equally.

Although the upper bounds on our estimates of current N_e_ are substantially smaller than what has been reported previously for *S. lewini* in the EP [17] and for other large sharks elsewhere [20], the larger number of loci in our study should result in more accurate N_e_ estimates. However, an additional reason why we obtained smaller estimates of N_e_ is likely related to our use of a newer method (MSVAR) with an underlying demographic model that specifically includes changes in N_e_ over time; methods that assume a static population size across the entire coalescent history of the sample are expected to yield upwardly biased estimates if N_e_ has recently declined in the past.

Although MSVAR, IMa, and M-ratio tests suggested EP populations of *S. lewini* have declined, mismatch distributions (mtDNA) showed that most populations in this region also experienced expansion, with point estimates of time since expansion (t) ranging from 90,606 to 136,061 years. While these two results may seem contradictory – signals of both expansion and decline – mismatch distributions are robust to subsequent change in N_e_ for a long time after the initial expansion [55], [81] whereas the coalescent structure of more rapidly evolving microsatellites likely track more recent demographic events [82].

### Population differentiation and divergence

**Figure 5:**
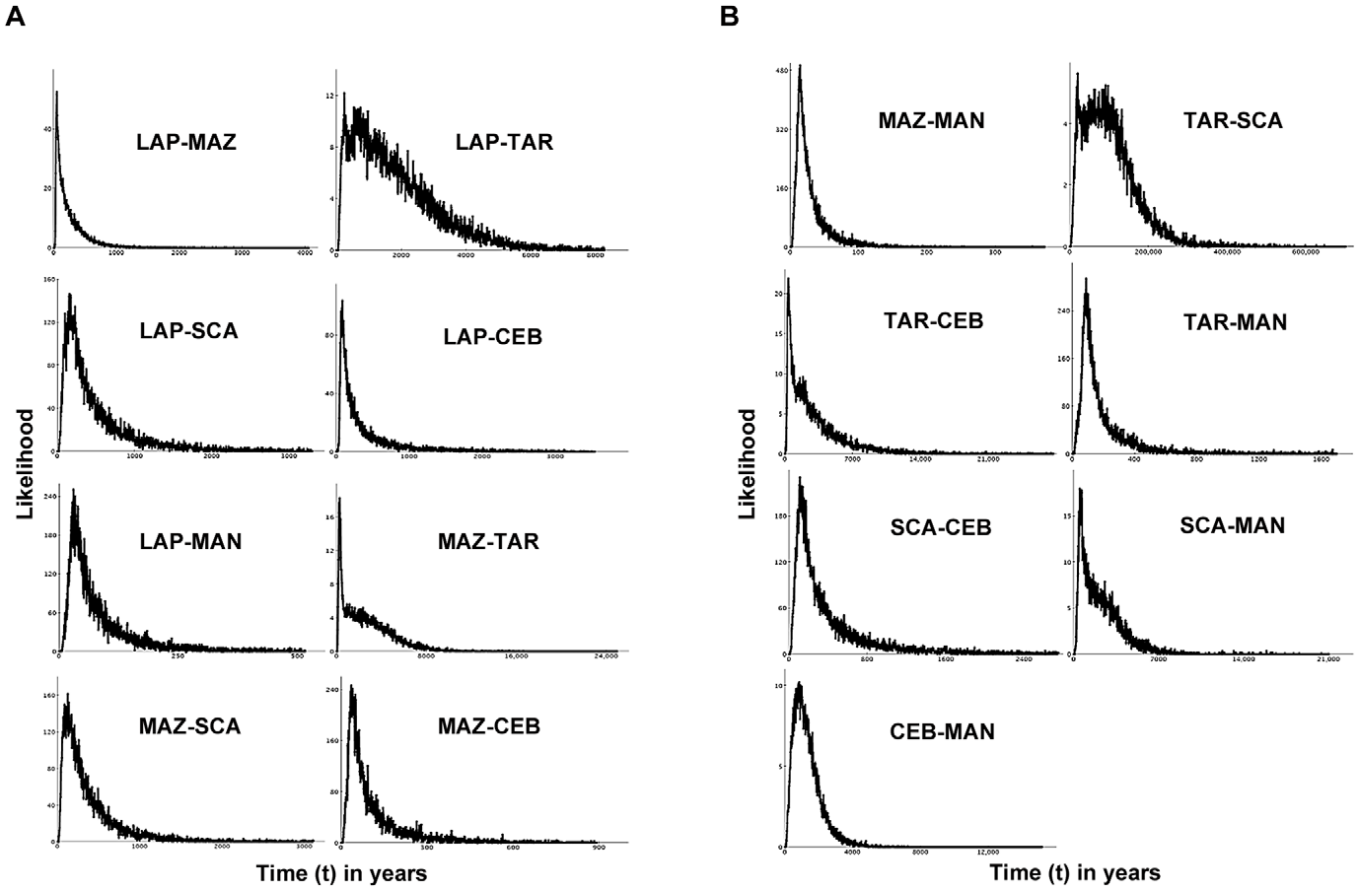
Posterior probability density of time since divergence for each population pair analyzed in IMa. The posterior probability density (PPD) of time (t), in years, is based on the fossil-calibrated substitution rate (6.03×10^−9^ subs/year).

Both nuclear and mitochondrial markers showed evidence of population subdivision: we found subtle but significant genetic differentiation among our sampled populations (global AMOVA for the microsatellites was statistically significant, *F*_ST_ = 0.005, *P*<0.001), most pairwise microsatellite *F*_ST_ estimates (between 0.002 and 0.014) were statistically significant, and both estimates of Φ_ST_ from mtDNA (r = 0.51, *P* = 0.05) and R_ST_ from microsatellite data (r = 0.42, *P* = 0.03) showed significant evidence of isolation by distance (IBD). Although the biological significance of subtle patterns of genetic differentiation as measured by *F*_ST_ can be difficult to evaluate on their own [83], [84], IMa posterior distributions for estimates of the time since population separation (t) had strong peaks (Fig. 5) and differed fundamentally from the uniform priors, with probabilities dropping to zero as t approached zero, indicating that each EP location is a sample from a separate population. Although the peaks in most of the PPDs for t were surprisingly recent (tens to hundreds of years), the upper bound on the 95% PPDs for 13 of 15 pairwise estimates of t fell within the Holocene. However, distributions were quite broad, indicating considerable uncertainty despite the use of 16 loci.

### Gene flow

Rates of gene flow (i.e., the number of migrants per generation, Nm = θ*m*/4) between EP populations inferred from IMa (ranging from 0.1 to 16.7 migrants per generation, Fig. 4) were less than 10% of point estimates of N_e_, suggesting the potential for demographic asynchrony [85], [86]. However, it is difficult to fully evaluate the demographic interdependence of populations without knowledge of population growth rates [87]. Nevertheless, an average of 2.4 migrants per generation probably provides little exogenous demographic input given the slow growth and maturation of *S. lewini*.

Because we did not sample all populations that could be exchanging migrants, our estimates of migration may be biased upwards. Simulations have shown that a third, unsampled population exchanging migrants with one of the two focal (sampled) populations considered in an IMa analysis will upwardly bias estimates of migration and θ [88]. Therefore, both connectivity and N_e_ may actually be lower than our data suggest. Gene flow from other regions is probably very low given that previous work ([17], Daly-Engel et al (unpublished data)) has shown that Nm based on both mtDNA and microsatellites from Hawaii and Indo-Pacific populations into Eastern Pacific populations is less than one. Therefore, given that the effects of ‘ghost populations’ are minimal when migration rates are low [89], and any upward bias in N_e_ would be evident in both current and ancestral estimates, it is unlikely that gene flow from central and western Pacific populations alone caused the large difference we observed between current and ancestral N_e_.

### Demographic history of *Sphyrna lewini* in the Eastern Pacific

Taken together, our data and analyses suggest that in the EP, *S. lewini* currently exists as a series of separate and potentially very small populations. Although low molecular diversity in sharks [32]–[35] is often attributed to low mutation rates in elasmobranchs [61], our analyses suggest that small N_e_ may be a significant factor contributing to low mtDNA (mean π = 0.0011, mean *h* = 0.5338; Table S3) and low microsatellite (mean *H_o_* = 0.770, mean *H_e_* = 0.792; *A_E_*<*A*, Table S2) diversity in *S. lewini*. However, it remains to be seen if small N_e_ is characteristic of other sharks with low levels of diversity. In addition to contributing to low diversity, small N_e_ could be an important evolutionary force driving population differentiation, rather than just restricted gene flow, given that some IMa estimates of gene flow were large enough (Nm>10) to maintain genetic homogeneity among populations [87].

### Potential factors contributing to population decline

Whether or not the decline in EP *S. lewini* is typical of other elasmobranchs, a history of decline following the last glacial maximum (LGM) 18,000–20,000 years ago in *S. lewini* is highly unusual considering warming after the LGM likely caused population expansions in many marine and terrestrial organisms [90]–[93]. Although precipitation decreased off the coast of Chile during the mid-Holocene (∼7700 – 4000 ybp; [94]), potentially reducing terrestrial run-off and nearshore productivity, mid-Holocene conditions in the equatorial EP were marked by increased upwelling and productivity [95]. Though these latter conditions are not expected to induce widespread decline in a marine apex predator, it is unclear how Holocene climate conditions affected coastal marine species throughout the EP. Another possibility is disease, which has been responsible for recent declines in several marine species [96]–[98]. However, little is known about the impacts of diseases in natural populations of sharks other than in general, they are thought to have robust immune systems [99]–[102].

An additional hypothesis is that prehistoric fishing practices initiated the decline [103], [104], which was later exacerbated by modern fishing. Archaeological remains from 14 sites along the EP coasts of Costa Rica, Panama, and Ecuador show that fishers using primitive nets and watercrafts as early as 6,000 years ago were catching sharks [105], with 3–5% of aboriginal middens comprised of shark remains (Richard Cooke, pers. comm.). Although fish comprised over 50% of edible meat remains in some places [106] and large inshore schools of juvenile hammerheads (typically in shallow embayments) are particularly vulnerable to even the simplest fishing methods, the extent of the potential impact of prehistoric fishing remains difficult to evaluate [107]–[111].

Considering alternative hypotheses regarding the cause for decline in EP *S. lewini* depends on the timing of decline as estimated with MSVAR, which is contingent on the rate and model of microsatellite mutation. It is difficult to speculate whether our prior range (including the known rates of bony fish and the slow end of the range of mammals) is too fast or too slow, which would either downwardly bias or upwardly bias our estimates of the time (t) since the start of decline, respectively. Departures from the simple SSM model of microsatellite evolution might also upwardly bias estimates of t from MSVAR because large mutations (addition or deletion of >1 repeat unit) will be modeled as a series of single steps [112]. However, a recent simulation study showed that MSVAR is robust to moderate departures from the SSM model [113], and this method has detected recent declines among mouse lemur populations (500 years before present (ybp); [114]) and among giant pandas (1000 ybp; [82]). Thus, while this method is capable of detecting change in N_e_ initiating as early as the Holocene, serious consideration of possible causes for decline will require further refinement of the timing of this demographic event as inferred from genetic data.

### Conclusions

Our use of coalescent methods to estimate current and historic N_e_ based on data from 16 independent loci suggests that scalloped hammerheads may have been far more abundant in the past than they are today. Low levels of genetic diversity in EP *S. lewini* may be a consequence of small N_e_, and genetic drift, rather than restricted gene flow, may be an important force causing population divergence. Our use of non-equilibrium models, which enabled us to estimate past population parameters for a globally endangered shark, has shed light on this vulnerable species' demographic history, providing a deeper understanding of the processes that led to existing levels and patterns of genetic diversity.

## Supporting Information

Table S1 **Microsatellite statistics per locus, per population.** *A* = total number of alleles per locus across all populations. *A_E_* = effective number of alleles. *A_p_* = private alleles per locus, per population. *H_o_* = observed heterozygosity per locus, per population, and *H_e_* = expected heterozygosity per locus, per population, as calculated in Arlequin v. 3.11 [32]. P-values in bold were significant after sequential Bonferroni correction of alpha (α). Number next to population abbreviation indicates number of samples.
(PDF)

Table S2 **Diversity statistics for mtDNA per population.** Nucleotide (π) and haplotype (*h*) diversities, and neutrality statistics (Fu's *F_s_* and Fu and Li's D*) are shown. Though neither neutrality statistic was significant at α = 0.05, only samples TAR and SCA show an increase in new mutations with negative D* values.
(PDF)

Table S3 **Life history data used for generation time (** ***G*** **) estimates.** Values of l*i* (age-specific survival rates), b*i* (birth rates), and p*i* (probability of a gene being inherited from a parent of age *i*), for all age classes, *i*, used to calculate the mean age of breeding adults, *G* [55].
(PDF)

Table S4 **Log-likelihood ratio test (LLRT) results from IMa analyses.** For each adjacent population pair, the likelihood of a simpler, alternative model where θ1 = θ2 = θA is shown [log(P)], the degrees of freedom for the LLRT of the full and alternative model, results from the test (2LLR), and the probability of achieving the test statistic by chance under the null model (*P*-value) are shown. All alternative models where population size has not changed were rejected in favor of the full model at α = 0.05.
(PDF)
