# Supplementary material for: Demographic Processes Underlying Subtle Patterns of Population Structure in the Scalloped Hammerhead Shark, Sphyrna lewini
Source: PLoS One. 2011 Jul 14;6(7):e21459. doi: 10.1371/journal.pone.0021459 (PMC3137562; doi:10.1371/journal.pone.0021459)
Supplement: Table S1 — Microsatellite statistics per locus, per population. A = total number of alleles per locus across all populations. AE = effective number of alleles. Ap = private alleles per locus, per population. Ho = observed heterozygosity per locus, per population, and He = expected heterozygosity per locus, per population, as calculated in Arlequin v. 3.11 [32]. P-values in bold were significant after sequential Bonferroni correction of alpha (α). Number next to population abbreviation indicates number of samples. (PDF) [file pone.0021459.s001.pdf]

Table S1. Microsatellite statistics per locus, per population. *A* = total number of alleles per locus across all populations. A_E = effective number of alleles. *Ap* = private alleles per locus, per population. H_o = observed heterozygosity per locus, per population, and H_e = expected heterozygosity per locus, per population, as calculated in Arlequin v. 3.11 [32]. P-values in bold were significant after sequential Bonferroni correction of alpha (α). Number next to population abbreviation indicates number of samples.

	LAP = 24						MAZ = 38						TAR = 40						SCA = 46					
**Locus**	***A***	***A E***	***Ap***	***Ho***	***He***	***p-val***	***A***	***A E***	***Ap***	***Ho***	***He***	***p-val***	***A***	***A E***	***Ap***	***Ho***	***He***	***p-val***	***A***	***A E***	***Ap***	***Ho***	***He***	***p-val***
Cli12	18	9.796	0	0.870	0.918	0.177	17	11.571	0	1.000	0.946	0.877	24	14.327	1	0.923	0.943	0.619	18	12.783	2	0.871	0.940	0.071
Cli100	9	4.82	0	0.792	0.809	0.619	8	5.684	0	0.868	0.834	0.724	9	6.317	0	0.825	0.811	0.465	9	4.477	2	0.767	0.806	0.230
SLE013	9	3.756	2	0.700	0.753	0.544	4	2.125	2	0.737	0.631	0.529	7	2.760	2	0.650	0.742	0.079	5	2.609	1	0.611	0.662	0.468
SLE025	13	7.121	0	0.778	0.884	0.108	14	6.644	3	0.946	0.891	0.788	19	6.099	0	0.825	0.881	0.155	13	6.811	6	0.841	0.875	0.388
SLE027	10	5.348	0	0.696	0.754	0.484	8	5.838	0	0.639	0.649	0.694	13	5.882	0	0.700	0.737	0.015	11	5.547	2	0.539	0.594	0.041
SLE028	6	3.615	0	0.909	0.832	0.223	7	3.803	1	0.694	0.838	0.202	9	4.071	1	0.950	0.834	0.031	8	3.767	1	0.852	0.842	0.264
SLE033	7	5.97	0	0.762	0.741	0.047	6	3.657	0	0.778	0.786	0.067	6	3.860	1	0.795	0.821	0.343	8	5.250	3	0.779	0.801	0.010
SLE038	8	3.806	0	0.800	0.854	0.616	5	2.359	1	0.667	0.746	0.799	10	2.807	0	0.700	0.800	0.399	8	2.800	1	0.764	0.823	0.022
SLE045	4	2.964	0	0.565	0.677	0.242	5	3.322	0	0.583	0.699	0.107	5	3.805	0	0.564	0.670	0.025	5	3.052	2	0.583	0.671	0.024
SLE053	8	3.216	0	0.435	0.704	0.006	7	3.482	0	0.639	0.790	**0.001**	9	5.959	0	0.725	0.832	0.320	11	6.452	1	0.727	0.872	**0.000**
SLE071	10	3.396	0	0.773	0.722	0.191	8	3.746	0	0.737	0.747	0.024	10	3.910	0	0.675	0.708	0.716	8	4.402	0	0.554	0.673	**0.002**
SLE077	18	9.68	0	0.864	0.918	0.623	14	7.364	2	0.895	0.882	0.367	20	8.545	3	0.947	0.917	0.811	15	8.699	4	0.820	0.868	0.138
SLE081	8	4.889	0	0.818	0.814	0.174	9	5.268	0	0.865	0.831	0.519	9	4.681	0	0.800	0.818	0.967	9	4.945	0	0.875	0.805	0.842
SLE086	5	3.639	0	0.773	0.742	0.499	5	3.620	0	0.730	0.732	0.683	5	2.623	1	0.795	0.680	0.028	6	4.272	0	0.631	0.749	**0.000**
SLE089	10	4.939	0	0.773	0.816	0.609	8	5.492	0	0.868	0.829	0.303	10	5.471	0	0.900	0.842	0.269	10	7.220	0	0.822	0.844	0.137

	CEB = 21						GPA = 9						MAN = 43					
**Locus**	***A***	***A E***	***Ap***	***Ho***	***He***	***p-val***	***A***	***A E***	***Ap***	***Ho***	***He***	***p-val***	***A***	***A E***	***Ap***	***Ho***	***He***	***p-val***
Cli12	30	15.15	0	0.810	0.944	0.044	12	10.125	0	0.667	0.954	**0.000**	30	14.948	1	0.940	0.249	**0.000**
Cli100	10	5.045	0	0.619	0.796	0.067	7	4.263	0	0.778	0.810	0.980	10	5.796	0	0.853	0.833	0.075
SLE013	11	2.927	0	0.714	0.632	0.642	5	3.306	0	0.556	0.739	0.775	15	2.781	5	0.583	0.645	0.045
SLE025	30	7.702	1	0.895	0.876	0.985	10	7.043	0	1.000	0.909	0.775	24	7.348	3	0.813	0.870	0.494
SLE027	21	6.109	0	0.619	0.659	0.016	7	4.909	1	0.750	0.767	0.845	15	5.113	1	0.671	0.658	0.694
SLE028	11	4.903	0	0.857	0.840	0.854	6	3.600	0	0.889	0.843	0.916	14	4.118	0	0.824	0.810	0.718
SLE033	12	5.493	0	0.667	0.756	0.400	6	5.586	0	0.889	0.765	0.832	9	4.524	0	0.735	0.763	0.343
SLE038	10	2.438	0	0.762	0.829	0.921	6	3.556	0	1.000	0.869	0.764	11	2.881	1	0.770	0.784	0.048
SLE045	7	2.999	0	0.762	0.689	0.735	5	3.447	0	0.444	0.752	0.127	5	3.211	0	0.662	0.693	0.199
SLE053	16	7.481	0	0.800	0.867	0.725	10	8.100	0	0.889	0.928	0.718	16	8.451	1	0.803	0.888	0.530
SLE071	9	3.026	0	0.789	0.794	0.740	6	2.700	0	0.667	0.667	0.677	13	3.379	2	0.675	0.709	0.390
SLE077	26	7.321	0	0.895	0.909	0.538	10	6.750	0	0.889	0.902	0.864	26	8.823	5	0.909	0.892	0.176
SLE081	10	5.022	1	0.947	0.819	0.567	6	4.765	0	0.667	0.837	0.722	9	5.196	1	0.800	0.813	0.515
SLE086	7	3.915	0	0.947	0.787	0.550	4	3.176	0	0.667	0.725	0.088	6	4.064	0	0.707	0.759	0.032
SLE089	11	6.238	1	0.895	0.885	0.758	8	5.063	0	0.778	0.850	0.845	12	6.021	0	0.808	0.839	0.508
