# Supplementary material for: Demographic Processes Underlying Subtle Patterns of Population Structure in the Scalloped Hammerhead Shark, Sphyrna lewini
Source: PLoS One. 2011 Jul 14;6(7):e21459. doi: 10.1371/journal.pone.0021459 (PMC3137562; doi:10.1371/journal.pone.0021459)
Supplement: Table S2 — Diversity statistics for mtDNA per population. Nucleotide (π) and haplotype (h) diversities, and neutrality statistics (Fu's Fs and Fu and Li's D*) are shown. Though neither neutrality statistic was significant at α = 0.05, only samples TAR and SCA show an increase in new mutations with negative D* values. (PDF) [file pone.0021459.s002.pdf]

Table S2. Diversity statistics for mtDNA per population. Nucleotide (π) and haplotype (h) diversities, and neutrality statistics (Fu's F_s and Fu and Li's $D*$) are shown. Though neither neutrality statistic was significant at $\alpha = 0.05$, only samples TAR and SCA show an increase in new mutations with negative $D*$ values.

Sample	n, mtDNA	π	h	F_s	$D*$
**LAP**	17	0.0008	0.4412	5.435	0.677
**MAZ**	22	0.0008	0.4156	5.709	0.064
**TAR**	20	0.0013	0.6158	4.087	-1.193
**SCA**	22	0.0013	0.5974	2.869	-1.729
**CEB**	18	0.0012	0.5686	4.196	-0.552
**GPA**	7	0.0010	0.5714	4.276	0.953
**MAN**	20	0.0010	0.5263	6.643	0.650
