# Supplementary material for: Demographic Processes Underlying Subtle Patterns of Population Structure in the Scalloped Hammerhead Shark, Sphyrna lewini
Source: PLoS One. 2011 Jul 14;6(7):e21459. doi: 10.1371/journal.pone.0021459 (PMC3137562; doi:10.1371/journal.pone.0021459)
Supplement: Table S3 — Life history data used for generation time ( G ) estimates. Values of li (age-specific survival rates), bi (birth rates), and pi (probability of a gene being inherited from a parent of age i), for all age classes, i, used to calculate the mean age of breeding adults, G [55]. (PDF) [file pone.0021459.s003.pdf]

Table S3. Life history data used for generation time (*G*) estimates. Values of l*i* (age-specific survival rates), b*i* (birth rates), and p*i* (probability of a gene being inherited from a parent of age *i*), for all age classes, *i*, used to calculate the mean age of breeding adults, *G* [see 55].

Age Class i	l_i	b_i	p_i	Age Class i	l_i	b_i	p_i
1	1.000	0.0	0.0	18	0.071	14.0	0.091
2	0.682	0.0	0.0	19	0.064	12.0	0.071
3	0.514	0.0	0.0	20	0.059	15.0	0.081
4	0.409	0.0	0.0	21	0.054	12.0	0.059
5	0.337	0.0	0.0	22	0.049	13.0	0.059
6	0.284	0.0	0.0	23	0.045	14.5	0.060
7	0.244	0.0	0.0	24	0.041	15.0	0.057
8	0.212	0.0	0.0	25	0.038	13.0	0.045
9	0.185	0.0	0.0	26	0.035	15.0	0.048
10	0.164	0.0	0.0	27	0.032	13.0	0.038
11	0.145	0.0	0.0	28	0.029	12.0	0.032
12	0.130	0.0	0.0	29	0.027	12.0	0.030
13	0.116	0.0	0.0	30	0.025	11.0	0.025
14	0.105	4.0	0.038	31	0.023	12.0	0.025
15	0.095	5.0	0.043	32	0.021	12.0	0.023
16	0.086	8.5	0.067	33	0.020	12.0	0.021
17	0.078	12.0	0.086				
