# Supplementary material for: Demographic Processes Underlying Subtle Patterns of Population Structure in the Scalloped Hammerhead Shark, Sphyrna lewini
Source: PLoS One. 2011 Jul 14;6(7):e21459. doi: 10.1371/journal.pone.0021459 (PMC3137562; doi:10.1371/journal.pone.0021459)
Supplement: Table S4 — Log-likelihood ratio test (LLRT) results from IMa analyses. For each adjacent population pair, the likelihood of a simpler, alternative model where θ1 = θ2 = θA is shown [log(P)], the degrees of freedom for the LLRT of the full and alternative model, results from the test (2LLR), and the probability of achieving the test statistic by chance under the null model (P-value) are shown. All alternative models where population size has not changed were rejected in favor of the full model at α = 0.05. (PDF) [file pone.0021459.s004.pdf]

Table S4. Log-likelihood ratio test (LLRT) results from IMa analyses. For each adjacent population pair, the likelihood of a simpler, alternative model where θ1 = θ2 = θA is shown [log(P)], the degrees of freedom for the LLRT of the full and alternative model, results from the test (2LLR), and the probability of achieving the test statistic by chance under the null model (*P*-value) are shown. All alternative models where population size has not changed were rejected in favor of the full model at α = 0.05.

Population Pair	Model	log(P)	d.f.	2LLR	*P* value
**LAP-MAZ**	$\theta_1=\theta_2=\theta_A$ m_1 m_2	-29.43	2	40.94	< 0.0001
	$\theta_1=\theta_2=\theta_A$ $m_1=m_2$	-20.38	3	22.84	< 0.0001
	$\theta_1=\theta_2=\theta_A$ $m_1=m_2=0$	-460.52	4*	903.11	< 0.0001
**LAP-TAR**	$\theta_1=\theta_2=\theta_A$ m_1 m_2	-460.52	2	904.88	< 0.0001
	$\theta_1=\theta_2=\theta_A$ $m_1=m_2$	-460.52	3	904.88	< 0.0001
	$\theta_1=\theta_2=\theta_A$ $m_1=m_2=0$	-460.52	4*	904.88	< 0.0001
**LAP-SCA**	$\theta_1=\theta_2=\theta_A$ m_1 m_2	-460.52	2	908.00	< 0.0001
	$\theta_1=\theta_2=\theta_A$ $m_1=m_2$	-460.52	3	908.00	< 0.0001
	$\theta_1=\theta_2=\theta_A$ $m_1=m_2=0$	-460.52	4*	908.00	< 0.0001
**LAP-MAN**	$\theta_1=\theta_2=\theta_A$ m_1 m_2	-460.52	2	907.06	< 0.0001
	$\theta_1=\theta_2=\theta_A$ $m_1=m_2$	-460.52	3	907.06	< 0.0001
	$\theta_1=\theta_2=\theta_A$ $m_1=m_2=0$	-460.52	4*	907.06	< 0.0001
**MAZ-TAR**	$\theta_1=\theta_2=\theta_A$ m_1 m_2	-460.52	2	904.40	< 0.0001
	$\theta_1=\theta_2=\theta_A$ $m_1=m_2$	-460.52	3	904.40	< 0.0001
	$\theta_1=\theta_2=\theta_A$ $m_1=m_2=0$	-460.52	4*	904.40	< 0.0001
**MAZ-SCA**	$\theta_1=\theta_2=\theta_A$ m_1 m_2	-460.52	2	907.67	< 0.0001
	$\theta_1=\theta_2=\theta_A$ $m_1=m_2$	-460.52	3	907.67	< 0.0001
	$\theta_1=\theta_2=\theta_A$ $m_1=m_2=0$	-460.52	4*	907.67	< 0.0001
**MAZ-CEB**	$\theta_1=\theta_2=\theta_A$ m_1 m_2	-460.52	2	906.90	< 0.0001
	$\theta_1=\theta_2=\theta_A$ $m_1=m_2$	-460.52	3	906.90	< 0.0001
	$\theta_1=\theta_2=\theta_A$ $m_1=m_2=0$	-460.52	4*	906.90	< 0.0001

Population Pair	Model	log(P)	d.f.	2LLR	*P* value
**MAZ-MAN**	$\theta_1=\theta_2=\theta_A$ m_1 m_2	-460.52	2	907.76	< 0.0001
	$\theta_1=\theta_2=\theta_A$ $m_1=m_2$	-460.52	3	907.76	< 0.0001
	$\theta_1=\theta_2=\theta_A$ $m_1=m_2=0$	-460.52	4*	907.76	< 0.0001
**TAR-SCA**	$\theta_1=\theta_2=\theta_A$ m_1 m_2	-460.52	2	909.13	< 0.0001
	$\theta_1=\theta_2=\theta_A$ $m_1=m_2$	-460.52	3	909.13	< 0.0001
	$\theta_1=\theta_2=\theta_A$ $m_1=m_2=0$	-460.52	4*	909.13	< 0.0001
**TAR-CEB**	$\theta_1=\theta_2=\theta_A$ m_1 m_2	-65.15	2	114.86	< 0.0001
	$\theta_1=\theta_2=\theta_A$ $m_1=m_2$	-69.59	3	123.74	< 0.0001
	$\theta_1=\theta_2=\theta_A$ $m_1=m_2=0$	-460.52	4*	905.59	< 0.0001
**TAR-MAN**	$\theta_1=\theta_2=\theta_A$ m_1 m_2	-460.52	2	909.77	< 0.0001
	$\theta_1=\theta_2=\theta_A$ $m_1=m_2$	-460.52	3	909.77	< 0.0001
	$\theta_1=\theta_2=\theta_A$ $m_1=m_2=0$	-460.52	4*	909.77	< 0.0001
**SCA-CEB**	$\theta_1=\theta_2=\theta_A$ m_1 m_2	-460.52	2	905.55	< 0.0001
	$\theta_1=\theta_2=\theta_A$ $m_1=m_2$	-460.52	3	905.55	< 0.0001
	$\theta_1=\theta_2=\theta_A$ $m_1=m_2=0$	-460.52	4*	905.55	< 0.0001
**SCA-MAN**	$\theta_1=\theta_2=\theta_A$ m_1 m_2	-350.98	2	687.5	< 0.0001
	$\theta_1=\theta_2=\theta_A$ $m_1=m_2$	-4.82	3	168.36	< 0.0001
	$\theta_1=\theta_2=\theta_A$ $m_1=m_2=0$	-91.42	4*	906.58	< 0.0001
**CEB-MAN**	$\theta_1=\theta_2=\theta_A$ m_1 m_2	-460.52	2	904.11	< 0.0001
	$\theta_1=\theta_2=\theta_A$ $m_1=m_2$	-460.52	3	904.11	< 0.0001
	$\theta_1=\theta_2=\theta_A$ $m_1=m_2=0$	-460.52	4*	904.11	< 0.0001

* Test distribution of 2LLR is a mixture
